# The Psychological Aspect of Hypnosis

**Published:** 1894-02

**Authors:** Wm. Romaine Newbold


### THE PSYCHOLOGICAL ASPECT OF HYPNOSIS.

BY WM. ROMAINE NEWBOLD, PH.D.[1]

Mr. President, Ladies, and Gentlemen,—In responding to your invitation to present to you the psychological aspect of hypnosis I find myself confronted by sundry difficulties. The phenomena in question are exceedingly complex, and it would be impossible to give an adequate review of them in the scanty time at my disposal. In the second place, they have engaged the attention of the scientific world but a few years, and many reported phenomena cannot be regarded as incontestably established. And in the last place, there is no consensus among psychologists as to the construction that is to be placed upon them.

The theories, psychological and physiological, that have been proposed to account for hypnosis are very nearly as numerous as the theorists, and not one has met with universal acceptance. Yet we are by no means relieved of the responsibility of framing hypotheses. Scarcely ever has a great scientific generalization been

---

[1] Lecturer on Philosophy, University of Pennsylvania.

established that was not constructed out of the ruins of a thousand hypotheses, and unless those thousand hypotheses had been devised, tested, and successively found wanting, we would never have had the material out of which to construct the true explanation. It is in this light that I regard the theory which I shall have the honor of putting before you to-night. It is based upon the facts of hypnosis chiefly as observed and reported by the school of Nancy, which my own limited experience has in every respect confirmed, and is most nearly akin to that of Dr. Despine. It is an effort to bring the facts of hypnosis into line with the psychological doctrine of the dynamic mental state, a doctrine which was, I believe, first taught clearly by Herbart, but for which a satisfactory scientific foundation has been but recently supplied by the researches of neurologists into the functions of the cerebral cortex. Scanty and disputable as our knowledge on these points is, there is a substantial agreement between the main results of introspective observation on the one hand and clinical observation and direct experiment on the other which is exceedingly encouraging.

You will readily understand that within the limits of this paper I can do no more than state and make plain the leading features of the tentative theory upon which I am now working. It is a theory that brings the chief phenomena of hypnosis into harmony with fundamental psychological principles and with the fundamental principles of cerebral physiology as far as I understand them, but which must undoubtedly suffer extensive modification as our knowledge of mind and brain progresses. I shall, however, in concluding, point out some of the dangers attending the practice of hypnotism and say a word as to the relation often supposed between hypnotism and telepathy.

It has long been known that certain individuals can be put by the use of definite agencies into a condition closely resembling sleep, but differentiated from it by sundry traits. These agencies are numerous, but all agree in involving the checking of the train of ideas, the restriction of attention to one idea or sensation, or to a constantly recurring but narrowly limited series of sensations, such as is given by passing the hands before the patient's eyes, by light touches or stroking, by rotating the head upon the shoulders, and so on. After the lapse of a time that varies in different individuals from a few seconds to thirty minutes or more, phenomena closely resembling sleep supervene. The eyelids become very heavy; they close; the field of consciousness becomes narrower and narrower, wanes, and finally disappears. When the patient awakes he believes that he has been asleep. But we can show that he has not been asleep in the ordinary sense of that word. He is nearly always cataleptic: his limbs tend to retain any position impressed upon them by the operator. He appears to be keenly alive to all that the operator says and does, but has neither eyes nor ears for any one else. This is the so-called phenomenon of *rapport*. He is frequently partially and sometimes totally analgesic. Pin-pricks and pinches are felt as touches, and burns as warmth. Sometimes, although not as often, he presents hyperæsthesia of sundry senses. He can read fine type at a considerable distance, can hear sounds that he cannot hear in the normal state, or can distinguish the compass points when very near together. But the most marked and most constant symptom is his absolute obedience to the word and wish of the operator. If told that he cannot open his eyes, cannot bend his arm, he cannot. If told to open his eyes, he opens them; if told that an elephant, a ghost, or anything else you please is before him, he sees it; and similar hallucinations can be produced in all the other senses. Negative hallucinations can also be produced. The operator can by a word destroy sight, hearing, touch, and other sensations, or can make the patient blind to one object or person, deaf to one sound or the voice of one person. Nor is it necessary that the suggestion be executed while the patient is in this abnormal condition. It may be executed hours, weeks, or even months afterwards. Sometimes the patient passes spontaneously while executing it into the condition in which he was while it was given, and he is then usually not aware that he has done it. Sometimes he does not so relapse, but executes it in his normal state, and then he will often devise all manner of plausible pretexts for his action,—anything, indeed, will he allege except the true reason, and that he rarely suspects. Finally, and this is a most characteristic symptom, no matter how deeply he appears to sleep, he can nearly always be awakened by a word. If I leave the patient undisturbed for a time while thus apparently asleep, all these phenomena usually disappear. He is no longer cataleptic, analgesic, hyperæsthetic, or suggestible; he is no longer *en rapport* with the operator, can no longer be awakened by the simple command; in other words, he is truly asleep in the ordinary sense of that word. Sometimes, however, if left to himself, he will awaken spontaneously, and no evil effects follow. He may feel a slight drowsiness, lassitude, or chilliness, such as we often feel when awakened from a nap, but nothing of a more serious nature need be feared.

I have described to you the hypnotic state in its most marked form,—that which is known as deep sleep, or lethargy. About twenty per cent. of all normal and healthy adults, if we accept the conservative estimate of Dr. Moll, can be put into this condition. Of the other eighty-odd per cent., about twenty or twenty-five per cent. are not at all or but little affected by the agencies which I have described. A slight drowsiness and weight in the eyelids they will probably feel, but not more. The remaining fifty-five or sixty per cent. are affected in various ways and to various degrees. Hardly will any two individuals present exactly the same phenomena, and scarcely any two writers agree upon the degrees that are to be recognized. Dessoir recognizes two, Charcot three, Forel three,—but his three do not coincide with those of Charcot,—Liébeault six, Chambard six, Bernheim nine, etc. Of these, the two proposed by Dessoir seem to me worthy of note. In the first grade only the power of originating voluntary movements is affected. Consciousness remains undisturbed, but the voluntary muscles are more or less withdrawn from voluntary control. The smaller and more highly specialized groups, such as those controlling eyes, lips, tongue, and fingers, are the first to be affected, then the larger groups. Suggestions affecting these muscles will then be executed, but hallucinations cannot be produced, nor will post-hypnotic suggestions be executed. In the second grade both the sensory and motor systems are affected and are subject to the suggestions of the operator. This is usually found in lethargy, but frequently without it. In the first grade only the functions connected with the efferent nerves are affected, in the second those connected with the afferent as well.

It is agreed by all observers that the most characteristic trait of hypnosis is the heightening of suggestibility. To this, catalepsy, automaton-like obedience, and the production of hallucinations can be reduced. Spontaneous analgesia and hyperæsthesia cannot be reduced to suggestibility unless the operator has carelessly dropped some remark in the presence of the patient indicating what he expects to find; yet in explaining suggestibility we shall, I think, find the clue to the explanation of these phenomena also.

Suggestibility is found in all normal individuals. Volumes have been written in illustration of the fact that the mere lodging of an idea in a man's mind is of itself a certain momentum tending to affect his action. It may take root and grow, or it may seem to have fallen upon barren soil and produce no effect. The world-wide phenomena of unconscious imitation, the influence which we unconsciously receive from our surroundings and exert upon one another, the curious epidemic manias of the Middle Ages, the periodic epidemics of crime, of which we have recently had several, all these are illustrations of the fact that the suggested idea is a momentum that has its own weight, which may indeed be overpowered, but which must always be taken into consideration. Now, in all degrees of hypnosis and in many conditions not classed as hypnotic the force of the individual idea is greatly exaggerated. In the most extreme cases it is no longer one of many momenta: it is the controlling power which seems to fatally determine what the man shall do, and even what he shall see and hear. Of such a man we say that his suggestibility has been heightened.

The explanation, therefore, of hypnosis, from the point of view of the psychologist, turns chiefly upon the explanation of suggestibility, and upon that we must for the present concentrate our attention. If suggestibility is characteristic of the normal man, its explanation must be found in the fundamental laws of mind and brain. We must then determine what conditions limit suggestibility in the normal state, and in what way we may remove these conditions and heighten it.

I must now ask your indulgence for a few minutes as I leave the problem of hypnotism proper and endeavor to sketch the main outlines of a psychological theory or method which we find to-day very wide-spread among neurologists, and rapidly gaining ground among those psychologists who bring to their proper work some acquaintance with physiology and pathology. According to this view all mental states are accompanied and conditioned by physical processes in the cerebral cortex. Upon this there is substantial agreement, and it may be regarded as the fundamental postulate of physiological psychology. Its converse, that every cortical process is accompanied by a mental state, is still disputed. If we admit this fundamental postulate, we are compelled to admit its corollary also,—that all the laws that govern mental states must admit of expression in physiological terms. And this corollary is fully borne out by the facts, as far as we are able to observe them. We must, then, learn to look upon the mental state precisely as we look upon all physical things. Scientific research in the physical world resolves itself into an inquiry into the causes and effects of phenomena, and the case is not otherwise in the mental world. The mental state must have its causes and its effects as certainly as the processes with which it is connected must have their causes and their effects. Its causes and effects need not form clearly distinguishable portions of the warp and woof of our conscious life. They may lie in the dim region that we call subconscious; but they exist, and, if our knowledge were exact, we could reason to them deductively with infallible certainty. Now, when we examine our mental states narrowly, we find that even in all the complexity of consciousness we can frequently trace out their causes and effects. Let us examine a few of these cases.

Every mental state is susceptible of development. Its dimmest and least developed form is that of the nascent idea just emerging into consciousness. Its most developed form is found in the vivid sensation which is qualitatively like that idea. This intensity is primarily determined by the intensity of the central or centripetal stimulus by which the corresponding cortical process is awakened; but it also depends upon the cerebral constitution of the individual and upon sundry other conditions.

Mental states have effects. Their ideational effects have long been known to psychologists, and studied under the name of association of ideas. I need do no more than allude to this most common phenomenon of subjective life. They have also motor effects. The relation of the vaso-motor system to the mental state is no new discovery. The blush of embarrassment, the pallor of fear, fainting at the sight of blood, are trite illustrations. Recent investigations have shown, however, that the connection between mental states and the distribution of the blood-supply is of the most thorough-going nature imaginable. Mental states also affect the voluntary muscles directly. To Alexander Bain belongs the honor, I believe, of having first established the relation of the motor idea to the production of the movement which it represents. The effect exerted by mental states upon the secretions of the skin, upon the salivary, mammary, and other glands, is a matter of daily observation; but their effect upon the processes of nutrition and repair is by no means as common or as universally admitted. Yet it seems to me that we have sufficient experimental as well as clinical evidence to warrant our admitting the existence of such effects, although we do not as yet understand the nervous mechanism by which they are produced or the conditions under which they operate.

Not only have mental states positive, they have also negative, effects. These negative effects are generally grouped under the term "inhibition." They are exceedingly obscure. The development of any mental state seems to affect unfavorably the development of simultaneously nascent but independent states,—a phenomenon commonly treated by psychologists under the title "passive attention." Individual sensory states sometimes seem to arrest one another directly, perhaps by a species of interference. Motor states can certainly check one another. Into these and kindred questions I shall not at present enter. I have, I hope, said enough to illustrate the fundamental law of which I speak,—that mental states, representing to us cortical processes of whose precise character we are wholly ignorant, must be regarded as belonging to the orderly system of nature quite as much as those cortical processes with which they are connected, and that we are entirely justified in ascribing to them those dynamic characteristics, those causes and effects which we ascribe to all physical phenomena. And into those characteristics, causes, and effects it is the business of the psychologist to inquire.

It is evident that in an inquiry into the causes of any such state or process we must go over very much the same ground as we have gone over in dealing with its effects. It may be caused *ab intra* by the operation of such antecedent states and processes as I have just described, or it may be caused *ab extra* by some sense perception. In general we term all states originated from without "suggestions," and all originated from within "auto-suggestions." Of all the agencies by which I can originate an idea in the mind of another none is as certain and as definite as language. Hence we commonly think of the suggested idea as having been suggested by words; but this is only accidental, not essential.

We have now completed our rapid survey of the dynamic mental state regarded as representing to us a cortical process. But it is evident that I have been presenting to you rather rare phenomena as illustrations of what I conceive to be one of the most fundamental laws of consciousness, and you may justly ask why this law, if it be ever operative, has so long escaped attention. The answer is not far to seek. If only our mental states existed singly, and each were free to develop itself and work out its own results without prejudice to or from other states, there would be little difficulty in determining what those results are. But in all normal states of consciousness the nascent mental state or cortical process finds itself in the midst of countless other subnascent, nascent, or definitely developed states, which it must modify, and by which it must suffer modification in countless and infinitely complex ways. It is, therefore, very difficult in the normal state to isolate any state sufficiently to distinguish its results from those of others.

Moreover, there exists in consciousness an activity of which the would-be scientific psychologist, anxious to bring all mental phenomena under mechanical or dynamic laws, usually says as little as he may. And certainly at our present stage of knowledge it is impossible to analyze it into any component factors or to determine the laws of its operation. Yet we cannot well ignore it, since it is the most constant and most powerful single factor of conscious life. This is what we call the consciousness of self. Sometimes it appears to be latent, and the man reacts upon the stimuli presented to him with almost fatal certainty. But then, again, it appears, and forthwith introduces into the train of ideas and movements an element of capriciousness that was not there before. When it is thus present and exerts its influence over our actions we are said to act voluntarily. The character of this conscious self is largely determined by hereditary influences. But it is also genetically related to the permanent factor in past experience, and may even be described as a function of that permanent factor, since any extensive changes in the latter are sooner or later followed by changes in the consciousness of self. We are, I think, justified in believing that in this at present mysterious inner activity we see the present conscious representative of the net aggregate result of our own past experience, and to some extent that of our ancestors, confronted with and acting upon the nascent mental state.

We are now in position to form a more clear conception of what is meant by suggestibility and to point out the chief factors that normally limit it. When we say that suggestibility is characteristic of all men, we mean that any mental state tends to develop to a certain degree and to produce certain definite results. This tendency is not, as a rule, very noticeable for two reasons: in the first place, any given mental state is but one of many, and we can rarely separate its results from those of others; and in the second, it is always exposed to modification by the most powerful and least understood element of consciousness,—the self. I have also tried in passing to indicate those physiological conceptions to which this psychological theory is most nearly related, and, if there be any truth in them, it is evident that the condition which we find in the normal man implies an inconceivably complex inter-correlation and functional co-ordination between the several portions of the cortex and between the organized systems of processes which we must conceive as existing within it.

Under what conditions, then, does suggestibility become heightened? Heightened suggestibility is found occasionally as a spontaneous phenomenon. Pierre Janet has, for example, a patient who is as suggestible in the waking state as most persons are in hypnosis.

She has never been hypnotized. It is sometimes found as one of the effects of certain drugs, among which are alcohol, ether, morphia, and chloroform. More light is much needed upon this question. Many dreams are due to the suggestions given by strong stimuli, which penetrate to the brain and arouse smouldering cortical processes, which pursue their devious way unhampered by the restraints of waking life, hence the frequently grotesque and unreal character of dreams. It is sometimes possible to cause a person to pass from normal sleep into a condition of heightened suggestibility that cannot be distinguished from hypnosis. An analogous state frequently occurs spontaneously, and is known as somnambulism. But of all the means of heightening suggestibility with which we are acquainted, none is as easy of application and certain in its effects as the concentration of attention and limitation of the conscious field; and those effects are, as far as the subject can describe them, precisely what our study of normal conditions has led us to postulate as the chief requisite to the production of heightened suggestibility. Sometimes the entire field of consciousness appears to fade away. Then we may suppose that the only things in consciousness are the isolated ideas which the operator suggests. In others the definite heterogeneity of normal life gives place to a dreamy state in which the vivid active self seems to have retired from the field. In others the self-consciousness remains, but finds itself so weakened that the commands of the operator appeal to it with irresistible power. This enfeeblement or destruction of the self is the most characteristic phenomenon of hypnosis, and is the chief reason for the heightened suggestibility. The nascent mental state finds the chief obstacle to its independent development removed, and we can study that development and its effects under new conditions.

We must not, however, assume that the development and effects of the mental state in hypnosis are what they are in normal life, or what they would be in normal life were we able to remove other influences without in any way modifying the intrinsic character of the state which we wish to study. The checking or stilling of self-consciousness is the most characteristic change of hypnosis, but it is by no means the only change; and the other changes which are found in different subjects are so numerous that it is impossible to describe them. One of the most common symptoms is what may be described as a species of mental inertia. An idea which in the normal state would awaken a mass of associated ideas, and thus give birth to a long train of ideas, will fall dead and inert in the hypnotized mind; it seems to have spent its energy in getting to existence. But occasionally the exact contrary is observed. A mere hint from the operator lodging in the sensitive brain of the subject will be spontaneously developed into a tragedy or comedy, as the case may be, and be acted out with a vivacity and power of which the normal individual would be incapable. In these and other personal peculiarities, such as the spontaneous analgesia and hyperæsthesia which I have mentioned, we may recognize variations in the distribution of cerebral activity of whose exact character we cannot form even a conception, and which doubtless rest upon organic and dynamic peculiarities in the cerebral constitution of the individual.

What cortical modification are we to conceive of as taking place in hypnosis? This is a question that is more easily asked than answered. I think we may say with some confidence that the normal cortical co-ordination of waking life is profoundly modified. This is indicated by the inhibition of the activity of the conscious self and the spontaneous alterations in sensation. But if we ask further just what portions of the cortex are chiefly concerned in this modification, it seems to me impossible even to propose a theory. We do not know whether the voluntary activity of the self is definitely related to some cortical area, as the various special senses are, or whether it is related to some cortical layer as supposed by some, or whether it is related to some organized system of processes distributed through the entire cortex. And as this is the psychical activity which is most profoundly modified in hypnosis, we may suppose that those portions of the cortex which are concerned with its manifestation are those chiefly modified in hypnosis. Yet, as I have shown, we have reason to suppose that other changes take place whose exact character we do not know at all. If, however, we ask to what other changes this modification of the processes connected with the manifestation of self-consciousness are most nearly related, I think we may say with some confidence that they are most nearly related to the changes that normally take place in sleep. In normal sleep the continuous ebb and flow of molecular change that accompanies waking life slowly and irregularly comes to a stop. Some little may remain in various portions of the brain, but it is for the most part in a state of comparative quiescence. In hypnosis we have reason to think that a similar stilling of the molecular activities begins to spread throughout the brain. In some way which we cannot now explain, the fixation of attention and voluntary limitation of the conscious field inhibits the activities connected with the conscious self first. If we leave the patient to himself these activities will either reassert themselves, or else the same change will spread on throughout the brain until the patient is in a normal sleep. And in the case of some persons this is the only effect that we can produce, by the use of the agencies which induce hypnosis in others. But if we do not leave the patient alone, but give him suggestions, we find that a large portion of the brain remains sensitive, that induced mental states or cortical processes—for some authorities contend that cortical processes in the hypnotized patient are not accompanied by any kind of consciousness—work out results which cannot be obtained in the patient while in the normal state. Hence hypnosis is in a sense a normal state artificially prolonged. It would, perhaps, be too much to say, with Mr. William James of Harvard, that we all pass through the hypnotic state every night while going to sleep. It seems to me probable that the methods which we use to produce hypnosis have a specific effect in determining the order in which the cortical activities are to sink into sleep, so to speak, which is not found in normal sleep. Yet the changes which take place in hypnosis probably do not essentially differ from those of normal sleep.

The phenomena of the hysterical condition, of epilepsy, of sundry forms of insanity, and of the rare trouble known as double consciousness, present many points of similarity with hypnosis, and we shall probably not be far wrong in classing all together as being due to derangement of cortical co-ordination. Sometimes, as, for example, in some forms of epilepsy, we have reason to believe that this disturbance is itself due to gross local lesions of the cortex. In many of these mental troubles we find derangements in self-consciousness and loss of voluntary control over different sensory or motor areas, and this is not seldom connected with heightened suggestibility. These facts have led many good authorities, notably Professor Charcot and the school of Paris, to regard hypnosis as a neurosis of the hysterical order depending upon some cerebral abnormality. I can therefore scarcely conclude this paper without making mention of this view and briefly indicating the reasons which lead me to dissent from it, although I do so with the modesty of the layman.

The researches of the school of Nancy have conclusively shown that the hypnotic state can be induced in the immense majority of normal adults, and they have been unable to discover any relation between susceptibility to hypnosis and hysteria, neurasthenia, or any other recognized disease. If, then, hypnosis is a disease, we are nearly all more or less tainted with it. This conclusion is supported by Moll, Dessoir, Wetterstrand, Forel, and many others. In the second place, I think that it is a misuse of language to term a state which can be experimentally induced and ended, but which does not spontaneously occur, and which, when thus experimentally produced and ended, does not tend to unfit the patient for the duties of life, a disease. But if ever we find hypnosis occurring spontaneously, it would tend to interfere with the patient's discharging his duties as a member of society, and in that case we would be justified in regarding it as a true neurosis of the hysterical order.

I have said that hypnosis may be regarded as an artificial prolongation of an approximately normal state. But, you may ask, what is the effect upon the patient of this artificial prolongation of a normal state? If the hypnotic state involves, as I am inclined to suppose, a temporary dissociation of cortical processes normally coordinated, even though this dissociation be produced by normal agencies, may it not be that by fixing and prolonging it beyond the normal time we may in a way loosen the bonds of union between the different areas of the cortex and produce a true hysterical neurosis? The question is most pertinent, and can be answered only by reference to observed facts. Some few cases of mental disturbance have been reported from the use of hypnosis, but such cases have been so few in number that we are justified in saying that the danger of such results in the case of the healthy patient in the hands of a trained operator is practically negligible. Some slight physical discomfort, as headache, drowsiness, and lassitude, are frequently found, but can usually be removed by suggestion. Headache is a very common symptom. But the only danger which is both serious and common appears to be the production of increased susceptibility to the hypnotic state. This is a very real danger, and we must ever be on the watch against it and cease experimenting at the first suggestion of it. Most authorities believe that we can always guard against it by suggestion. We can to some degree, but I know of no facts that show that the suggestion will last as long as the increased susceptibility to hypnosis.

Apart from the physical dangers springing from the use of hypnosis, we must consider the moral. That moral dangers exist there can be no question. Already several cases have been reported in which criminal assault has been committed by the aid of hypnosis, and the French experimenters have enticed patients to commit imaginary murders and thefts, to sign promissory notes, and to bear false witness in the same way. Yet I do not feel that our present knowledge justifies much apprehension on this score.

We hear much in the literature of hypnotism of the cases in which the suggestions succeed, but little of those in which they fail. And they very often fail, as I shall hope to show you to-night. A suggestion whose execution the patient would find repugnant in the waking state will often meet with great resistance and sometimes fail of execution. The influence of the operator over his patient is much greater than in waking life, but it is perhaps never absolutely complete, and if there are persons who could be induced by hypnotic suggestion to commit crimes from which they would shrink while awake, such persons are probably exceedingly rare and their weakness is rarely suspected. One other danger remains which I must not fail to mention, and that is the danger springing from careless suggestions. I know a gentleman who thoughtlessly told his patient that the devil was after him, and had great ado to save the terrified patient from throwing himself from a sixth story window. The operator must ever remember that his every word is caught by the patient, no matter how impassive the latter may seem, and he must always be on his guard against saying anything of an exciting or terrifying character.

I have never seen any good reason for believing that there is any relation between hypnosis and telepathy. It is true that telepathic phenomena are from time to time reported as having been observed in hypnosis, but such phenomena are also reported in waking life. Most certainly the hypnotized patient is not, as a rule, sensitive to telepathic influences. But even if we admit that he sometimes is, it would not essentially modify the theory which I have outlined, and we would not be compelled to return to the theory of a force emanating from the operator and controlling the subject. We would merely be compelled to admit the possibility of a suggestion being conveyed in some way and by some means not at present understood.

In conclusion, let me say a word or two on the practical principles that should guide us in the use of hypnosis. No one should attempt it without having thoroughly studied the question, acquainted himself with the works of the great authorities, and having had some experience with an instructor. Its use should be limited to purposes of research, instruction, and therapeutics. A witness should always be present. The operator must rigidly respect the personal rights of the patient. He must have his full and intelligent consent to the experiments proposed, and must never endeavor to obtain influence over him at times or in ways other than those agreed upon. He must guard the patient effectually from any future inconvenience arising from increased susceptibility, and if he have reason to doubt his ability so to guard him, he must stop his experiments. And in the last place, and this principle includes all the others, he must ever have in mind and at heart the true welfare of his patient, and act in accordance with it.

